# Bioprocessing strategies for cost-effective simultaneous removal of chromium and malachite green by marine alga *Enteromorpha intestinalis*

**DOI:** 10.1038/s41598-020-70251-3

**Published:** 2020-08-10

**Authors:** Ragaa A. Hamouda, Noura El-Ahmady El-Naggar, Nada M. Doleib, Amna A. Saddiq

**Affiliations:** 1 Department of Biology, Faculty of Sciences and Arts Khulais, University of Jeddah, Jeddah, Saudi Arabia; 2 Microbial Biotechnology Department, Genetic Engineering and Biotechnology Research Institute, University of Sadat City, Sadat City, Egypt; 3 Department of Bioprocess Development, Genetic Engineering and Biotechnology Research Institute, City of Scientific Research and Technological Applications, Alexandria, Egypt; 4 Department of Microbiology, Faculty of Applied and Industrial Science, University of Bahri, Khartoum, Sudan; 5 Department of Biology, Faculty of Sciences, University of Jeddah, Jeddah, Saudi Arabia

**Keywords:** Biochemistry, Biotechnology, Environmental sciences

## Abstract

A large number of industries use heavy metal cations to fix dyes in fabrication processes. Malachite green (MG) is used in many factories and in aquaculture production to treat parasites, and it has genotoxic and carcinogenic effects. Chromium is used to fix the dyes and it is a global toxic heavy metal. Face centered central composite design (FCCCD) has been used to determine the most significant factors for enhanced simultaneous removal of MG and chromium ions from aqueous solutions using marine green alga *Enteromorpha intestinalis* biomass collected from Jeddah beach. The dry biomass of *E. intestinalis* samples were also examined using SEM and FTIR before and after MG and chromium biosoptions. The predicted results indicated that 4.3 g/L *E. intestinalis* biomass was simultaneously removed 99.63% of MG and 93.38% of chromium from aqueous solution using a MG concentration of 7.97 mg/L, the chromium concentration of 192.45 mg/L, pH 9.92, the contact time was 38.5 min with an agitation of 200 rpm. FTIR and SEM proved the change in characteristics of algal biomass after treatments. The dry biomass of *E. intestinalis* has the capacity to remove MG and chromium from aquatic effluents in a feasible and efficient manner.

## Introduction

In recent years, contamination of the environment by heavy metals and dyes becomes a major area of concern. Cobalt, chromium, nickel and copper are used in the textile industry to fix dyes, which causes environmental problems. Chromium is used in textile industry as a catalyst in the dyeing process and as an oxidant in the wool textile processing^1–3^. Recently, heavy metals and dyes are used in many industries like, energy and fuel production, leather tanning, etc. All these industries discharge large quantities of toxic wastes directly or indirectly into the environment with untreated effluents which cause a serious environmental pollution and endangering human life^4–6^.

It is well known that heavy metals induced acute or chronic toxicity, carcinogenicity and induce multiple organ damage of skin, bladder, liver and lungs^7^. One of the greatest challenges for researchers is to reduce the toxicity of heavy metals especially in developing countries. Companies are avoiding the management of industrial wastes owing to their massive costs, which could increase environmental pollution from huge quantities of a potentially dangerous waste of heavy metals^8^.

Each metal has its unique physico-chemical characteristics that lead to its particular toxicological mode of action^9^. Chromium (Cr) has commonly two oxidation states; Cr^+3^ and Cr^+6^; both states are stable, predominantly in the environment^10^. Chromium is an essential element, considered as a micronutrient in humans, plays an important role in glucose, fat, protein and cholesterol metabolism. At higher concentration, it has a toxic effect for humans, animals and plants. Due to the extensive use of Cr in a wide range of industries^11,12^; the consequent environmental pollution of chromium has increased, causing the greatest concern in the last recent years^13^.

According to the World Health Organization (WHO), the maximum permissible limits of Cr(VI) in drinking water is 0.05 mg/L^14^. Chromium cannot be biodegraded easily and therefore chromium exceeds the permissible limit accumulated in the food chain and become destructive to human health. Hexavalent chromium, Cr(VI) was reported to be relatively more harmful compared to Cr(III)^7^. Exposure to chromium compounds can cause allergenicity and carcinogenicity in humans and in animals^15^. Ingestion of any significant amount of chromium cause mouth and nasal septum ulcers, kidney failure, abdominal pain, vomiting, indigestion, acute tubular necrosis, induce DNA damage and even death^7,16^.

Malachite green (MG) dye (Supplementary Fig. S1) is widely used in various industrial fields as dyeing, distilleries, fungicide and also antiseptic to control parasites and disease of fish^17–19^. Malachite green is a hazardous material, difficult to remove and causes environmental problems, genotoxicity, histopathological and biochemical alterations in aquatic organisms^17^. Malachite green causes tumours to many human organs as lungs, breast and ovary, damage heart, liver, spleen and kidney^20,21^. Malachite green bind to DNA and can lead to DNA damage and induce the formation of DNA adducts^17^.

There are different conventional treatment technologies for removing contaminants of heavy metals from the environment or wastewater effluents and reduction of heavy metal toxicity. Some of these treatments are Physico-chemical removal processes include: chemical reduction, ion exchange, adsorption on activated carbon, membrane filtration, chemical precipitation and electrochemical removal^22^. But most of these conventional processes are of limited application that is due to their significant disadvantages, which include high cost, high energy consumption, low selectivity and generate large amounts of toxic wastes or incomplete removal^23^. It is therefore necessary to use cheap, safe and more effective methods to remove heavy metals from wastewater^24^.

Biosorption technique has become one of the most promising technology and alternative potential technique for treatment of wastewater and removal of heavy metals to be below concentration limits established by regulatory authorities^25^. Biosorption involves the use of biological material such as living organisms, mainly microorganisms (algae, yeasts, fungi and bacteria) as biosorbents. The marine algae have been effectively used as biosorbents to remove numerous hazardous ingredients and potentially toxic elements^6^. Marine green algae are one of the most promising organisms with a high ability for heavy metals removal. It has many advantages because of (1) sustainable, biodegradable and conveniently accessible throughout the year, (2) large surface area and quick accumulation of metal, (3) availability of various binding sites on their surface, (4) have a high binding capacity for metals, (5) little or no need for harmful chemicals, (6) algal nutritional requirements are minimal and do not generate toxic substances^26,27^.

The mechanism involved in the biosorption process by marine green algae relies on the presence of different functional groups of the biomacromolecules like lipids, polysaccharides and proteins on the algal cell wall surface^28,29^. These functional groups (e.g. sulfhydryl, phosphate, carboxyl, thiol and amino groups) serve as adsorption sites^30^. The metal ions are typically adsorbed to the algal cell wall surface through the physical and/or chemical adsorption or ion exchange between the metal cations and the cell surface. Malachite green dye can be absorbed from aqueous solution by macro green alga *Enteromorpha*^31^. Dry biomasses of some algae such as *Cladophora glomerata*, *Enteromorpha intestinalis* and *Microspora amoena* have been used as biosorbent to eliminate Cr(VI) from aqueous solutions. Al-Homaidan et al*.*^32^ compared between various biosorbent including *Enteromorpha* for the removal of Cr (VI), they reported that the *Enteromorpha* was the best effective biosorbent for hexavalent chromium ions from aqueous solution.

Optimization of biosorption processess can be performed by using the classical method in which it only can vary one factor at one time while the other factors are maintained at constant levels. The traditional approach has drawbacks because it is tedious, hard, and consumes more chemicals and time because a great number of experiments are required to determine the optimum conditions at each time. Moreover, it doesn't reflect the impact of interaction between the independent factors^33^. Meanwhile, these limitations can be excluded by optimization using Face-centered central composite design (FCCCD). FCCCD was used to determine the significant effects of the process factors on simultaneous removal of chromium and MG from aqueous solutions using *E. intestinalis* dry biomass. FCCCD is a set of mathematical and statistical techniques that can be used to maximize and to study the interaction effects of several factors at one time. FCCCD is faster, more economical, reduces the number of the experiments, effective and define the most optimal conditions and maintain good accuracy of the expected response compared to the classical method.

The current study aimed to assess the biosorption efficacy of marine green alga, *E. intestinalis*, biomass for simultaneously decolourization of malachite green and chromium ions removal from aqueous solutions. The statistical optimization for simultaneously chromium ions removal and malachite green decolourization has also performed. SEM and FTIR were used for biomass characterization before and after chromium and malachite green biosorption.

## Results and discussion

The biosorption processes are complicated systems and their performance is greatly affected by various physico-chemical process parameters such as pH, temperature, etc. In this study, the effects of five factors, namely biomass of *E. intestinalis* as a biosorbent, the concentration of chromium ions, the concentration of MG dye, initial pH level and the contact time on the removal efficiency of chromium ions and MG dye (as responses) were evaluated.

### Statistical optimization of chromium and MG removal by *E. intestinalis* biomass

A total number of fifty experimental trials of FCCCD (Table 1) were used to evaluate the impacts of five process variables and to determine their optimal levels for simultaneous removal of chromium and MG from aqueous solutions using *E. intestinalis* dry biomass. Experimental and predicted results of chromium ions and MG removal are shown in Table 1. The results show significant differences in the percentages of chromium and MG removal by *E. intestinalis* based on the variation of the five variables. Depending on the observed data attained; chromium removal percent varied significantly from 47.14 to 89.98% and in the malachite green removal ranged from 7.79 to 97.39. The highest levels of chromium (89.98%) and MG (97.39%) removal were obtained in the run no. 32 when the malachite green concentration was 6 mg/L, chromium concentration was 120 mg/L, algal biomass was 3 g/L, initial pH level was 10 and the incubation time was 40 min. While the minimum chromium (47.14%) and malachite green (7.79%) removal obtained in the run no. 2 when the malachite green concentration was 2 mg/L, chromium concentration was 120 mg/L, algal biomass was 3 g/L, initial pH level was 7 and the incubation time was 40 min.

**Table 1:** FCCCD matrix used for simultaneous adsorption of malachite green and chromium ions by using *E. intestinalis.*

Std	Run	Type	X_1_	X_2_	X_3_	X_4_	X_5_	Malachite green removal (%)			Chromium removal (%)		
								Actual	Predicted	Residuals	Actual	Predicted	Residuals
32	1	Fact	1	1	1	1	1	89.56	90.26	− 0.70	83.88	84.16	− 0.28
33	2	Axial	− 1	0	0	0	0	7.79	10.22	− 2.43	47.14	46.85	0.29
12	3	Fact	1	1	− 1	1	− 1	87.13	92.52	− 5.39	86.00	86.16	− 0.16
17	4	Fact	− 1	− 1	− 1	− 1	1	10.93	5.95	4.98	53.37	52.69	0.67
45	5	Center	0	0	0	0	0	81.70	77.10	4.61	72.74	73.82	− 1.08
22	6	Fact	1	− 1	1	− 1	1	89.13	89.62	− 0.49	85.78	84.18	1.60
19	7	Fact	− 1	1	− 1	− 1	1	4.58	9.48	− 4.90	55.02	53.60	1.42
37	8	Axial	0	0	− 1	0	0	55.37	58.83	− 3.46	70.61	70.51	0.10
25	9	Fact	− 1	− 1	− 1	1	1	9.67	11.54	− 1.87	53.58	55.20	− 1.62
39	10	Axial	0	0	0	− 1	0	85.01	90.42	− 5.41	82.36	84.14	− 1.79
8	11	Fact	1	1	1	− 1	− 1	84.23	79.85	4.38	80.49	80.32	0.16
50	12	Center	0	0	0	0	0	77.82	77.10	0.72	74.14	73.82	0.32
38	13	Axial	0	0	1	0	0	55.82	58.07	− 2.26	68.79	68.99	− 0.20
42	14	Axial	0	0	0	0	1	82.33	85.31	− 2.98	67.62	68.94	− 1.32
47	15	Center	0	0	0	0	0	79.98	77.10	2.88	73.94	73.82	0.12
30	16	Fact	1	− 1	1	1	1	86.74	90.19	− 3.45	82.50	83.28	− 0.78
21	17	Fact	− 1	− 1	1	− 1	1	10.61	8.13	2.48	48.58	49.71	− 1.14
9	18	Fact	− 1	− 1	− 1	1	− 1	24.19	29.79	− 5.60	63.07	63.97	− 0.89
27	19	Fact	− 1	1	− 1	1	1	27.88	28.02	− 0.14	64.81	63.25	1.56
2	20	Fact	1	− 1	− 1	− 1	− 1	86.58	85.85	0.73	75.74	75.17	0.58
24	21	Fact	1	1	1	− 1	1	79.36	76.74	2.62	79.65	77.92	1.73
48	22	Center	0	0	0	0	0	77.42	77.10	0.32	74.07	73.82	0.25
23	23	Fact	− 1	1	1	− 1	1	8.50	8.97	− 0.47	48.44	49.70	− 1.26
31	24	Fact	− 1	1	1	1	1	28.46	28.59	− 0.14	53.70	54.37	− 0.66
3	25	Fact	− 1	1	− 1	− 1	− 1	44.30	41.27	3.03	67.95	68.40	− 0.45
28	26	Fact	1	1	− 1	1	1	83.89	79.80	4.09	75.75	76.41	− 0.66
46	27	Center	0	0	0	0	0	80.22	77.10	3.12	74.77	73.82	0.95
7	28	Fact	− 1	1	1	− 1	− 1	24.26	26.59	− 2.34	56.93	56.68	0.25
18	29	Fact	1	− 1	− 1	− 1	1	75.67	77.55	− 1.88	69.73	70.52	− 0.80
26	30	Fact	1	− 1	− 1	1	1	78.11	77.03	1.07	75.33	74.60	0.72
11	31	Fact	− 1	1	− 1	1	− 1	59.91	55.25	4.65	76.79	77.58	− 0.79
40	32	Axial	0	0	0	1	0	97.39	97.70	− 0.30	89.98	88.29	1.69
1	33	Fact	− 1	− 1	− 1	− 1	− 1	30.12	28.76	1.36	61.80	61.92	− 0.12
35	34	Axial	0	− 1	0	0	0	71.03	75.27	− 4.24	76.96	76.05	0.91
16	35	Fact	1	1	1	1	− 1	85.07	88.82	− 3.75	86.47	86.10	0.37
4	36	Fact	1	1	− 1	− 1	− 1	83.89	84.63	− 0.74	75.61	75.40	0.22
14	37	Fact	1	− 1	1	1	− 1	84.63	79.77	4.86	78.83	79.65	− 0.82
29	38	Fact	− 1	− 1	1	1	1	16.13	14.80	1.33	48.76	47.24	1.52
5	39	Fact	− 1	− 1	1	− 1	− 1	15.13	16.78	− 1.65	52.47	51.14	1.34
34	40	Axial	1	0	0	0	0	66.32	69.60	− 3.28	67.98	68.37	− 0.39
41	41	Axial	0	0	0	0	− 1	93.26	95.99	− 2.73	75.75	74.53	1.22
49	42	Center	0	0	0	0	0	84.72	77.10	7.62	74.14	73.82	0.32
10	43	Fact	1	− 1	− 1	1	− 1	84.40	80.77	3.62	79.71	78.78	0.93
20	44	Fact	1	1	− 1	− 1	1	67.79	67.36	0.43	64.48	65.19	− 0.70
6	45	Fact	1	− 1	1	− 1	− 1	81.63	83.76	− 2.13	79.29	81.01	− 1.72
13	46	Fact	− 1	− 1	1	1	− 1	19.77	18.89	0.87	47.81	48.20	− 0.39
43	47	Center	0	0	0	0	0	76.92	77.10	− 0.18	75.07	73.82	1.25
15	48	Fact	− 1	1	1	1	− 1	42.48	41.66	0.82	61.16	60.89	0.27
44	49	Center	0	0	0	0	0	80.86	77.10	3.76	72.07	73.82	− 1.75
36	50	Axial	0	1	0	0	0	80.09	81.56	− 1.47	78.71	79.72	− 1.01

Variable	Variable code	Coded and actual levels											
		− 1	0	1									
Malachite green conc. (mg/L)	X_1_	2	6	10									
Chromium conc. (mg/L)	X_2_	40	120	200									
Algal biomass (g/L)	X_3_	1	3	5									
Initial pH level	X_4_	4	7	10									
Incubation time (min)	X_5_	20	40	60									

### Multiple regression analysis and ANOVA

The results of FCCCD for removal of malachite green by *E. intestinalis* biomass were analyzed by multiple regression statistical analysis and ANOVA (analysis of variance) calculations which are tabulated in Table 2. Statistical regression analysis parameters such as determination coefficient (R^2^) value, predicted R^2^ value, adj R^2^ value, *F*-value and lack of fit have been determined and evaluated for the model reliability.

**Table 2:** Analysis of variance for adsorption of malachite green by *E. intestinalis* obtained by FCCCD.

Source of variance		Degrees of freedom	Sum of square	Mean of square	*F*-value	*P*-value	Coefficient estimate
Overall model		20	43,277.76	2,163.89	127.64	< 0.0001*	77.10
Linear effect	X_1_	1	29,967.77	29,967.77	1767.73	< 0.0001*	29.69
	X_2_	1	336.13	336.13	19.83	0.0001*	3.14
	X_3_	1	4.90	4.90	0.29	0.5948	− 0.38
	X_4_	1	450.05	450.05	26.55	< 0.0001*	3.64
	X_5_	1	970.12	970.12	57.23	< 0.0001*	− 5.34
Interaction effect	X_1_X_2_	1	376.50	376.50	22.21	< 0.0001*	− 3.43
	X_1_X_3_	1	195.72	195.72	11.55	0.0020*	2.47
	X_1_X_4_	1	74.46	74.46	4.39	0.0449*	− 1.53
	X_1_X_5_	1	421.37	421.37	24.86	< 0.0001*	3.63
	X_2_X_3_	1	14.51	14.51	0.86	0.3625	− 0.67
	X_2_X_4_	1	335.70	335.70	19.80	0.0001*	3.24
	X_2_X_5_	1	161.12	161.12	9.50	0.0045*	− 2.24
	X_3_X_4_	1	2.35	2.35	0.14	0.7125	0.27
	X_3_X_5_	1	401.18	401.18	23.66	< 0.0001*	3.54
	X_4_X_5_	1	41.52	41.52	2.45	0.1284	1.14
Square effect	X_1_^2^	1	3,420.41	3,420.41	201.76	< 0.0001*	− 37.19
	X_2_^2^	1	4.30	4.30	0.25	0.6184	1.32
	X_3_^2^	1	860.06	860.06	50.73	< 0.0001*	− 18.65
	X_4_^2^	1	711.39	711.39	41.96	< 0.0001*	16.96
	X_5_^2^	1	454.27	454.27	26.80	< 0.0001*	13.55
Error effect	Lack of fit	22	444.75	20.22	3.02	0.0688	
	Pure error	7	46.88	6.70			
R^2^	0.9888	Std. dev	4.12				
Adj R^2^	0.9810	Mean	60.78				
Pred R^2^	0.9642	C.V. %	6.77				
Adeq Precision	34.38	PRESS	1568.50				

*Significant values, *F* Fishers's function, *P* Level of significance, *C.V* Coefficient of variation.

A regression model with a value of R^2^ exceeding 0.9 was considered strongly correlated^34^. The current R^2^ value of the model used for malachite green removal by *E. intestinalis* (R^2^ = 0.9888) reflects that 98.88% of variance in malachite green removal were assigned to the used factors and the model cannot explain just 1.22 per cent of the total variance. In addition, the Adj R^2^ value of the malachite green removal % (Adj R^2^ = 0.9810) was high also to verify the great model significance (Table 2). The value of predicted R^2^ of 0.9642 agreed with the value of the Adj R^2^. This indicates a strong correlation between the experimental and predicted values of the malachite green removal percentages. A relatively small value of the coefficient of variation % (C.V. = 6.77%) reflects high precision and accuracy of the experiments values^35^. The current model's adequate precision value is 34.38; the PRESS (predicted residual sum of squares) value is 1568.50. The Std. Dev. (standard deviation) and mean values of the malachite green model are 4.12 and 60.78; respectively (Table 2). Here, the ANOVA for the malachite green removal % indicate that the model terms are highly significant which is confirmed by the *F* (Fishers’ variance ratio) value (*F-*value = 127.64) and a very small *P-*value [˂ 0.0001] (Table 2). *P-*value less than 0.05 indicate that the terms of the model are significant^36^. The lack of fit for malachite green removal % is not significant (*F-*value = 3.02; *P-*value = 0.0688) (Table 2).

Data were interpreted by means of the signs of the coefficients (negative or positive impact on the response) and *P-*value (*P* < 0.05) for understanding the interactions between test variables. Two-factor interactions can appear as an oppositional (negative) or complementary (positive) effect. The significance value of coefficients can indicate that the linear coefficients of X_1_, X_2_, X_4_ and X_5_ are highly significant together with the interaction effects between X_1_X_2_, X_1_X_3_, X_1_X_4_, X_1_X_5_, X_2_X_4_, X_3_X_5_, X_2_X_5_, X^2^_1_, X^2^_3_, X^2^_4_ and X_5_^2^. In addition, the *P-*value of coefficients (*P-*value < 0.05) can indicate that the interactions between X_1_ and X_2_; X_1_X_5_; X_3_X_5_ had a very significant impact on malachite green decolourization by *E. intestinalis*. The linear coefficients of X_3_, interactions between X_2_X_3_, X_3_X_4_ and X_4_X_5_ and X_2_ quadratic effect are nonsignificant model terms that do not make a significant contribution to the malachite green removal.

The fit summary results seen in Supplementary Table S1 indicate that the quadratic polynomial model is the highest significant model and sufficient to fit the FCCCD of malachite green removal by *E. intestinalis* where the terms are significant (*P-*value < 0.0001) with non-significant lack of fit (*P-*value = 0.0688; *F*-value = 3.02). The quadratic model summary data indicate the lower Std. Dev. value (4.12) and higher values of the adjusted and predicted R^2^ (0.9810 and 0.9642; respectively).

The polynomial regression equation of second order for malachite green removal by *E. intestinalis* (Y) can be written according to the coefficients that were fitted as the following:

1 $$ \begin{aligned}{\text{Y}} &= {77}.{1}0 + {29}.{\text{69X}}_{{1}} + {3}.{\text{14X}}_{{2}} {-}0.{\text{38X}}_{{3}} + {3}.{\text{64X}}_{{4}} {-}{5}.{\text{34X}}_{{5}} {-}{3}.{\text{43X}}_{{1}} {\text{X}}_{{2}} + {2}.{\text{47X}}_{{1}} {\text{X}}_{{3}} {-}{1}.{\text{53X}}_{{1}} {\text{X}}_{{4}} +_{{}} {3}.{\text{63X}}_{{1}} {\text{X}}_{{5}} \\ &\quad{-}0.{\text{67X}}_{{2}} {\text{X}}_{{3}} + {3}.{\text{24X}}_{{2}} {\text{X}}_{{4}} {-}{2}.{\text{24X}}_{{2}} {\text{X}}_{{5}} + 0.{\text{27X}}_{{3}} {\text{X}}_{{4}} + {3}.{\text{54X}}_{{3}} {\text{X}}_{{5}} + {1}.{\text{14X}}_{{4}} {\text{X}}_{{{5} + }} {37}.{\text{19X}}_{{1}}^{{2}} + {1}.{\text{32X}}_{{2}}^{{2}} {-}{18}.{\text{65X}}_{{3}}^{{2}} \\ &\quad+ {16}.{\text{96X}}_{{4}}^{{2}} + {13}.{\text{55X}}_{{5}}^{{2}}\end{aligned} $$

where Y is the predicted value of malachite green removal % by *E. intestinalis* biomass. X_1_-X_5_ are coded values for the concentration of malachite green, chromium concentration, *E. intestinalis* biomass concentration, initial pH level and contact time.

Similarly, the results of FCCCD for chromium ions removal % by *E. intestinalis* biomass were analyzed by multiple regression statistical analysis and ANOVA (analysis of variance) calculations which are tabulated in Table 3. The current R^2^ value of the model = 0.9928, the Adj R^2^ value of 0.9878 and predicted R^2^ of 0.9754 were large to validate the model's high significance (Table 3). The current model's adequate precision value is 49.34; the PRESS (predicted residual sum of squares) value is 166.15 and the percentage of coefficient of variation value is 1.86%. The Std. Dev. (standard deviation) and mean values of the chromium model are 1.30 and 69.81; respectively (Table 3). Here, the ANOVA of the quadratic regression model for the chromium ions removal % verify that the model terms are highly significant which is confirmed by the *F* (Fishers’ variance ratio) value (*F-*value = 199.94) and a very small *P-*value [˂ 0.0001] (Table 3). The lack of fit for chromium ions removal % is not significant (*F-*value = 1.91; *P-*value = 0.1927) (Table 3).

**Table 3:** Analysis of variance for adsorption of chromium by *E. intestinalis* obtained by the FCCCD.

Source of variance		Degrees of freedom	Sum of square	Mean of square	*F*-value	*P*-value	Coefficient estimate
Overall model		20	6,714.68	335.73	199.94	< 0.0001*	73.82
Linear effect	X_1_	1	3,936.38	3,936.38	2,344.29	< 0.0001*	10.76
	X_2_	1	115.02	115.02	68.50	< 0.0001*	1.84
	X_3_	1	19.60	19.60	11.67	0.0019*	− 0.76
	X_4_	1	145.84	145.84	86.86	< 0.0001*	2.07
	X_5_	1	265.00	265.00	157.82	< 0.0001*	− 2.79
Interaction effect	X_1_X_2_	1	77.90	77.90	46.39	< 0.0001*	− 1.56
	X_1_X_3_	1	553.45	553.45	329.61	< 0.0001*	4.16
	X_1_X_4_	1	4.94	4.94	2.94	0.0971	0.39
	X_1_X_5_	1	42.06	42.06	25.05	< 0.0001*	1.15
	X_2_X_3_	1	1.70	1.70	1.01	0.3222	− 0.23
	X_2_X_4_	1	102.03	102.03	60.77	< 0.0001*	1.79
	X_2_X_5_	1	61.97	61.97	36.90	< 0.0001*	− 1.39
	X_3_X_4_	1	49.62	49.62	29.55	< 0.0001*	− 1.25
	X_3_X_5_	1	121.89	121.89	72.59	< 0.0001*	1.95
	X_4_X_5_	1	0.43	0.43	0.26	0.6162	0.12
Square effect	X_1_^2^	1	649.80	649.80	386.99	< 0.0001*	− 16.21
	X_2_^2^	1	40.90	40.90	24.36	< 0.0001*	4.07
	X_3_^2^	1	41.00	41.00	24.42	< 0.0001*	− 4.07
	X_4_^2^	1	380.06	380.06	226.34	< 0.0001*	12.40
	X_5_^2^	1	10.73	10.73	6.39	0.0172*	− 2.08
Error effect	Lack of fit	22	41.74	1.90	1.91	0.1927	
	Pure error	7	6.95	0.99			
R^2^	0.9928	Std. dev	1.30				
Adj R^2^	0.9878	Mean	69.81				
Pred R^2^	0.9754	C.V. %	1.86				
Adeq precision	49.34	PRESS	166.15				

*Significant values, *F*: Fishers's function, *P* level of significance, *C.V* coefficient of variation.

The significance value of coefficients can indicate that all the linear and quadratic coefficients are significant. The coefficients *P-*values also indicate that between the five factors studied, two-factor interactions between X_1_, X_2_ (MG conc. and chromium conc.), X_1_X_3_ (MG conc. and algal biomass conc.), X_1_X_5_ (MG conc. and incubation time), X_2_X_4_ (chromium conc. and initial pH ), X_2_X_5_ (chromium conc. and incubation time), X_3_X_4_ (algal biomass and initial pH) and X_3_X_5_ (algal biomass and incubation time ) had a very significant effects on chromium removal by *E. intestinalis.* On the other hand, the interactions between X_1_X_4_; X_2_X_3_; X_4_X_5_ are no significant model terms that do not make a significant contribution to the removal of chromium ions.

The fit summary results seen in Supplementary Table S2 show that the quadratic polynomial model is the highest significant and sufficient to fit the FCCCD of chromium ions removal by *E. intestinalis* where the terms are significant (*P-*value < 0.0001) and lack of fit is not significant (*P-*value = 0.1927; *F*-value = 1.91).

The polynomial regression equation of second order for chromium ions removal by *E. intestinalis* (Y) can be written according to the coefficients that were fitted as the following:

2 $$ \begin{aligned}{\text{Y}} &= \, + {73}.{82} + {1}0.{\text{76X}}_{{1}} + {1}.{\text{84X}}_{{2}} {-}0.{\text{76X}}_{{3}} + {2}.0{\text{7X}}_{{4}} {-}{2}.{\text{79X}}_{{5}} {-}{1}.{\text{56X}}_{{1}} {\text{X}}_{{2}} + {4}.{\text{16X}}_{{1}} {\text{X}}_{{3}} + 0.{\text{39X}}_{{1}} {\text{X}}_{{4}} \\ &\quad+ {1}.{\text{15X}}_{{1}} {\text{X}}_{{5}} {-}0.{\text{23X}}_{{2}} {\text{X}}_{{3}} + {1}.{\text{79X}}_{{2}} {\text{X}}_{{4}} {-}{1}.{\text{39X}}_{{2}} {\text{X}}_{{5}} {-}{1}.{\text{25X}}_{{3}} {\text{X}}_{{4}} + {1}.{\text{95X}}_{{3}} {\text{X}}_{{5}} + 0.{\text{12X}}_{{4}} {\text{X}}_{{5}} {-}{16}.{\text{21X}}_{{1}}^{{2}} \\ &\quad+ {4}.0{\text{7X}}_{{2}}^{{2}} {-}{4}.0{\text{7X}}_{{3}}^{{2}} + {12}.{4}0{\text{X}}_{{4}}^{{2}} {-}{2}.0{\text{8X}}_{{5}}^{{2}}\end{aligned} $$

where Y is the predicted value of chromium ions removal % by *E. intestinalis* biomass. X_1_-X_5_ are coded values for the concentration of malachite green, chromium concentration, *E. intestinalis* biomass concentration, initial pH level and contact time.

### Three dimensional (3D) plots for malachite green removal

The 3D graphs are tools to understand the interactions between the process factors and to predict the optimal conditions for the highest percentage of malachite green removal. 3D graphs for the five variables combined in pairs “X_1_ X_2_, X_1_ X_3,_ X_1_ X_4,_ X_1_ X_5,_ X_2_ X_3,_ X_2_ X_4,_ X_2_ X_5,_ X_3_ X_4,_ X_3_ X_5,_ and X_4_ X_5_” were constructed by plotting the percentages of malachite green removal on Z-axis versus two independent process factors while maintaining the other independent process factors at their center levels.

**Figure 1:**
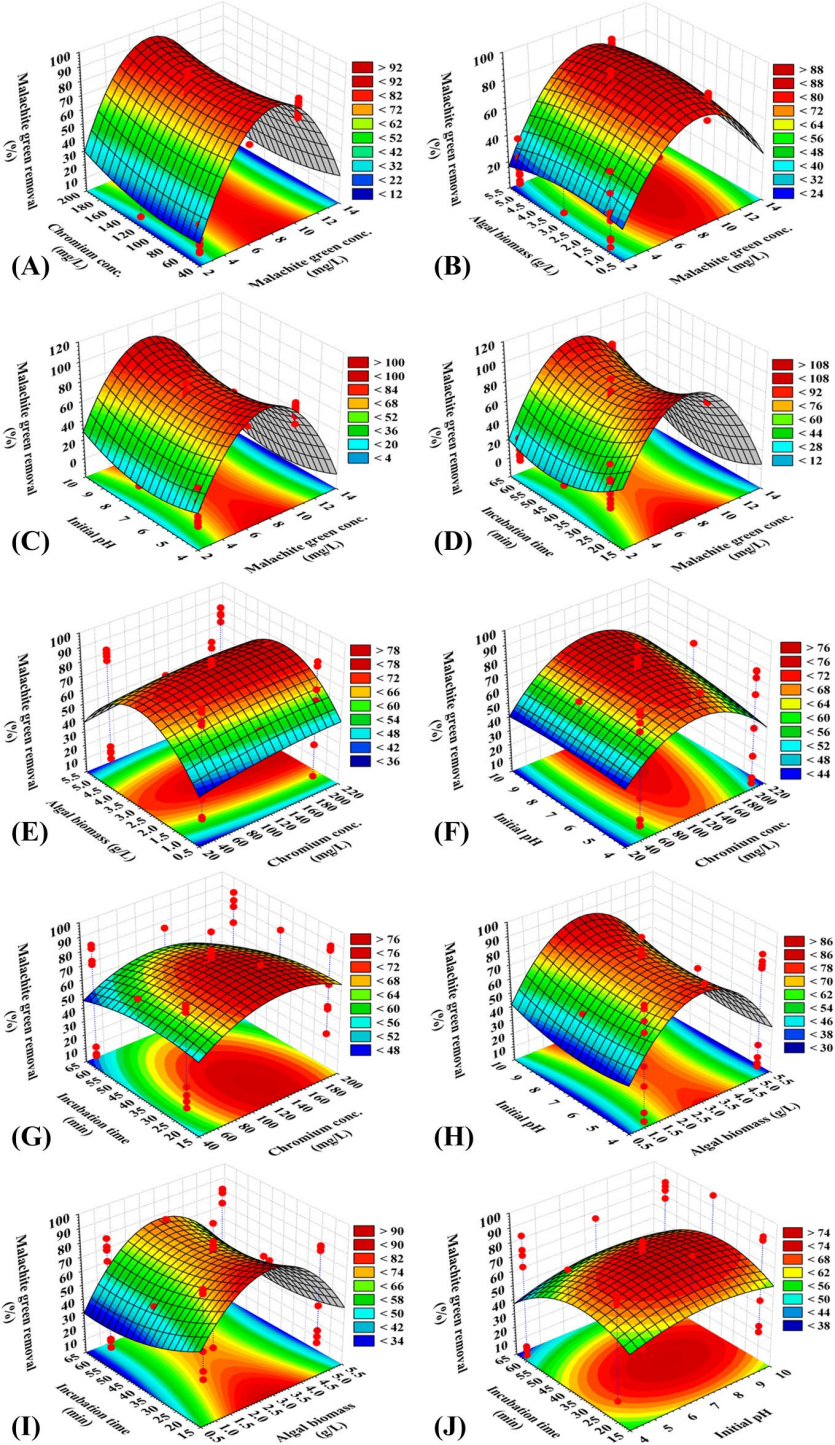
Three-dimensional surface plot of for adsorption of malachite green by *E. intestinalis*, showing the interactive effects of two variables at a time of the five tested variables. The three-dimensional surface plots were created by using statistical software package, STATISTICA software (Version 8.0, StatSoft Inc., Tulsa, USA).

The 3D graph (Fig. 1A), shows the impact of malachite green concentration (X_1_) and chromium concentration (X_2_) on the percentage of malachite green removal, whereas *E. intestinalis* biomass concentration (X_3_), initial pH (X_4_) and incubation time (X_5_) were maintained their center levels. Figure 1A indicates that the highest percentage of malachite green removal is obviously located close to the central level of malachite green concentration. Furthermore, the lower and higher concentrations of malachite green (X_1_) resulted in lower malachite green removal percentages. By analyzing Fig. 1A and solving the Eq. (1), the maximum predicted value for malachite green removal of 97.70% could be attained at the optimal predicted levels of malachite green and chromium concentrations of 10 and 200 mg/L; respectively by using *E. intestinalis* biomass concentration of 3 g, initial pH 7 and 40 min incubation time.

The 3D graph (Fig. 1B), showing the effects of malachite green concentrations (X_1_) and *E. intestinalis* biomass concentrations (X_3_) on the percentage of malachite green decolourization, at center levels of chromium concentrations (X_2_), initial pH (X_4_) and incubation time (X_5_). Figure 1B indicates that the highest percentage of malachite green removal was attained by using 3 g/L *E. intestinalis* biomass concentration, after which the decolourization of malachite green decreased. The lower and higher concentrations of malachite green (X_1_) resulted in low percentage of malachite green decolourization and the highest percentage of malachite green removal obviously located at center levels of malachite green. By analyzing Fig. 1B and solving the Eq. (1), the maximum predicted malachite green removal of 97.07% could be attained at the optimal predicted levels of malachite green and *E. intestinalis* biomass concentrations of 6 mg/L and 3 g/L; respectively by using chromium concentrations of 120 mg/L, initial pH 7 and 40 min contact time.

The 3D graph (Fig. 1C), showing the effects of two factors, malachite green concentrations (X_1_) and initial pH level (X_4_), on malachite green removal percentage, while the other factors (chromium concentrations, *E. intestinalis* biomass concentration and contact time) were kept at their center levels. The percentage of malachite green removal increased gradually with increasing levels of malachite green concentrations to the central level, after which the malachite green removal decreased. On the other hand, 3D graph (Fig. 1C), indicates that the high levels of initial pH increased malachite green decolourization. By analyzing Fig. 1C and solving the Eq. (1), the maximum predicted malachite green removal of 97.7% could be attained at the optimal predicted levels of 6 mg/L malachite green (X_1_) and pH 8 by using 120 mg/L chromium concentration, 3 g/L *E. intestinalis* biomass concentration and 40 min contact time.

The 3D graph (Fig. 1D), showing the effects of malachite green concentrations (X_1_) and contact time (X_5_) on the malachite green decolourization efficiency, when the chromium concentrations (X_2_), *E. intestinalis* biomass concentration (X_3_) and initial pH (X_4_) were kept at their center levels. By analyzing Fig. 1D and solving the Eq. (1), the maximum predicted malachite green removal of 97.7 percent could be attained at the optimal predicted levels of 6.5 mg/L malachite green (X_1_) and contact time (X_5_) of 45 min by using 120 mg/L chromium concentration, 3 g/L *E. intestinalis* biomass concentration and pH 7.

The 3D plots (Fig. 1E–G) represent the effects of chromium concentrations (X_2_) and algal biomass (X_3_) (Fig. 1E); chromium concentrations (X_2_) and pH (X_4_) (Fig. 1F); chromium concentrations (X_2_) and contact time (X_5_) (Fig. 1G) on the malachite green decolourization efficiency, when the other independent variables were kept at their center levels. Figure 1E–G shows that the lower and higher levels of chromium concentrations, algal biomass and contact time led to a low percentage of malachite green removal while, the higher level in pH support increase in the malachite green decolourization.

The three-dimensional response surface curves in Fig. 1H,I indicates that the higher and lower levels of alga biomass increase the malachite green decolourization but the higher level of pH causes increase in malachite green decolourization. Figure 1J showed lower and higher levels of contact time decrease malachite green removal percentage and higher value of malachite green decolourization was obtained beyond high pH value.

### The adequacy of the model

**Figure 2:**
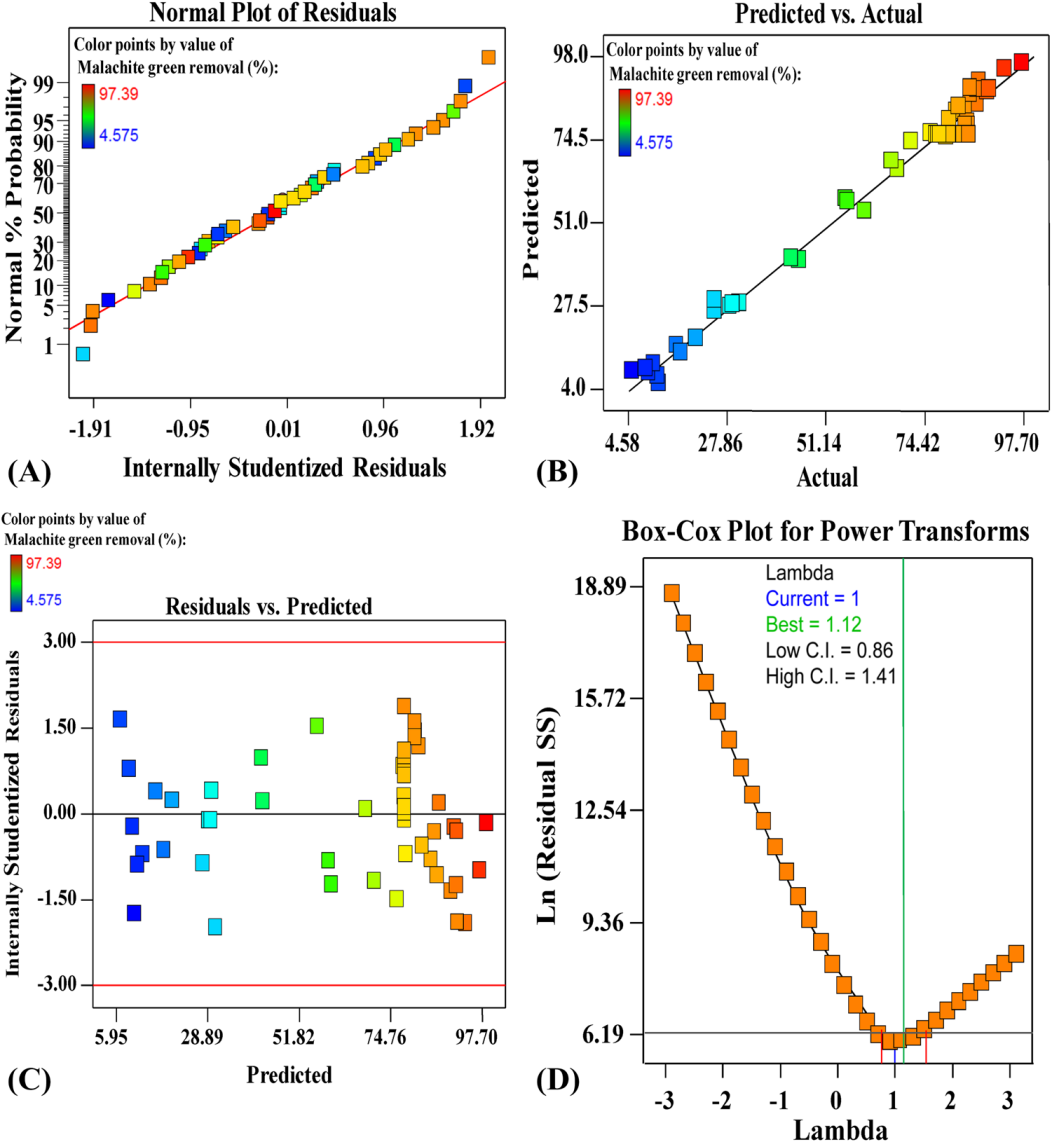
(**A**) Normal probability plot of internally studentized residuals, (**B**) plot of predicted versus actual, (**C**) plot of internally studentized residuals versus predicted values and (**D**) Box- Cox plot of model transformation, of malachite green adsorption. Image was created by using Design Expert version 7 for Windows software.

The normal probability plot is the graph that signifying the normal distribution of the residuals to validate the model suitability^37^. The residuals are the differences between the responses' experimental values and their predicted theoretical values. Low residual values indicate very accurate model prediction^38^. Figure 2A shows the studentized residuals plotted versus the normal probability for malachite green removal efficiency by *E. intestinalis* biomass. The residuals are normally distributed; they are located along the straight diagonal line of malachite green decolourization %. Therefore, the normal distribution of the residuals reveals the model's validity^39^. Figure 2B shows the actual versus predicted percentages for malachite green removal percentages from aqueous solution. Figure 2B displays all the points along the diagonal line, indicating that the model’s predicted percentages coincide with the actual percentages, confirming that the model is accurate. Figure 2C shows the studentized residual versus predicted values for malachite green removal percentages. Figure 2C in this study indicated that the residuals randomly distributed about zero line. This meant that the residuals had an almost constant variance over the variable ranges. Figure 2D shows Box-Cox plot of model transformation of malachite green removal percentages. As can be seen in Fig. 2D, the Lambda (λ) optimal value of 1 lies between the two vertical red lines so that no data transformation is required.

### Three dimensional (3D) plots for chromium removal

**Figure 3:**
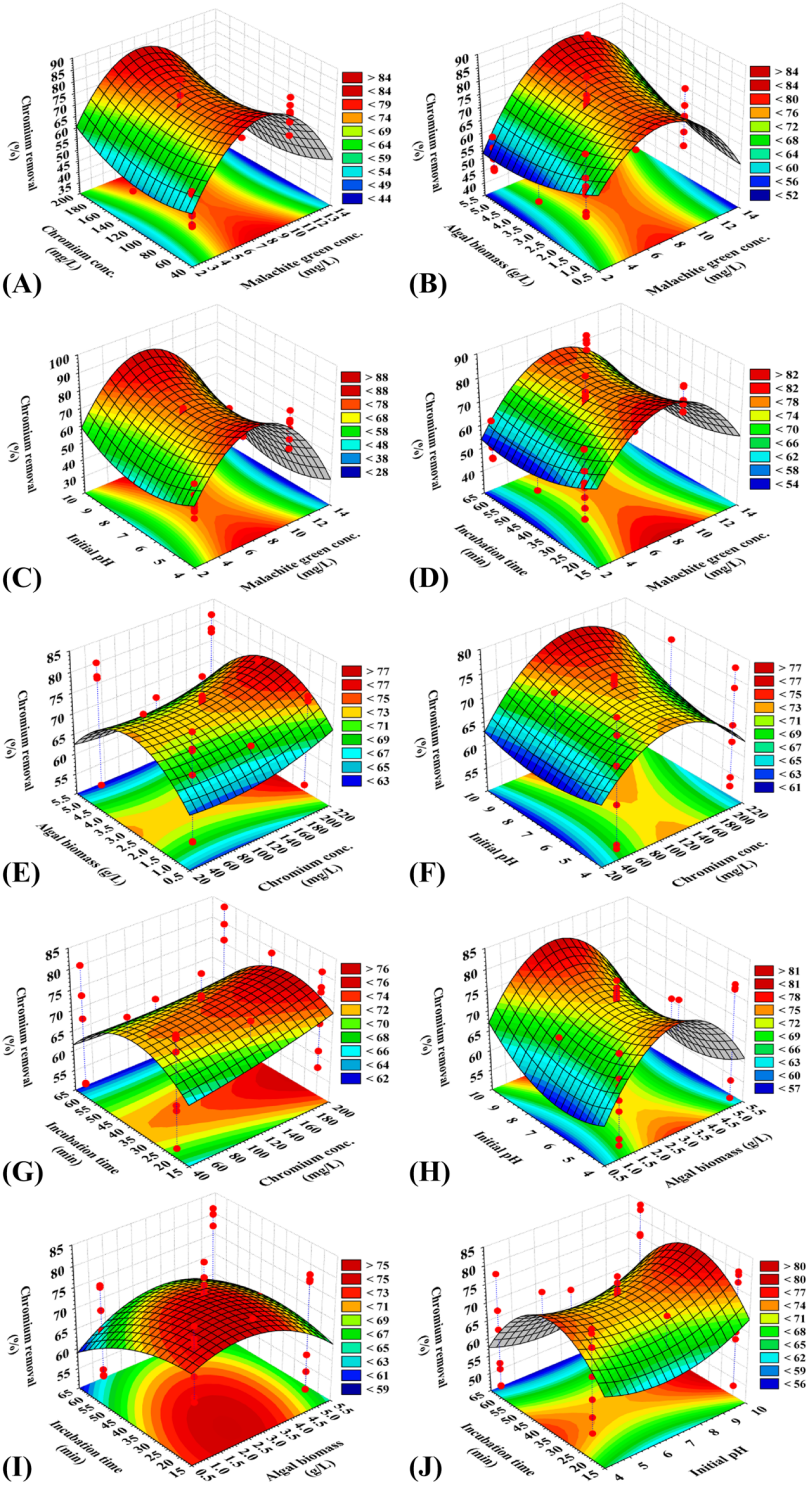
Three-dimensional surface plot of for biosorption of chromium by *E. intestinalis*, showing the interactive effects of two variables at a time of the five tested variables. The three-dimensional surface plots were created by using statistical software package, STATISTICA software (Version 8.0, StatSoft Inc., Tulsa, USA).

Figure 3 presents the three-dimensional plot for chromium removal percentages as a function of malachite green concentration, chromium concentrations, algal biomasses, initial pH level and incubation time. Figure 3A–D demonstrates that higher and lower levels of malachite green decrease the percentage of chromium removal from aqueous solutions and the maximum chromium removal percent attained at the middle level of malachite green. Figure 3A,B demonstrates that the lower and higher levels of algal biomass increase the chromium removal percentage; Fig. 3C, higher levels of pH and middle levels of malachite green concentrations causes an increase of chromium removal percentage. Figure 3D reveals that the contact time has a low effect the percentage of chromium removal.

The 3D plots obtained in Fig. 3E–G presents the effects of independent variable chromium concentrations and algal biomass (Fig. 3E); chromium concentrations and pH (Fig. 3F); chromium concentrations and contact time (Fig. 3G). The 3D plots indicated that chromium removal percentage increased at the central (zero) levels of biomass (Fig. 3E), at central levels of contact time (Fig. 3G), the high and low values of contact time resulted in a chromium removal decrease. Figure 3F shows that the maximum chromium removal % has been attained at the central level of chromium concentrations and at high pH level.

Figure 3H,I depicted that the effect of independent variable, algal biomass, contact time and pH, while the other variables were kept at their center levels. The percentage of chromium removal was decreased at low and high levels of algal biomass and contact time and increase with an increase in pH.

The removal of different dyes and metal ions increased with increasing the dye and metal ions solely or simultaneously and reached to highest. Further increasing in dye and metal ions concentrations leads to a slight increase in the removal percentage. This can be due to all active sites on the algal biomass adsorptive for metals ions and MG that free at the beginning resulting in high dye and metals ion adsorptions, so further increasing of MG and heavy metals ions resulting in decreasing of adsorption due to algal biomass free active site are few to binding with excess MG dye or metal ions, So dry algae can adsorbed heavy metals and dyes effectively, but it was most affected and limited by optimization of the required process factors such as temp., pH, algal biomass, as well as, the concentrations of dyes and heavy metals. Husien et al.^40^ reported that when the concentrations of pollutant increased the removal of pollutants was decreased due to the binding sites available were decreased on the surface of algae.

### Effect of chromium concentrations

The chromium concentrations are the most important factors that impact of chromium removal by algae, so chromium removal was decreased by increasing chromium concentrations due to the *Chlorella* cells was degraded^40,41^. Al-Homaidan et al.^32^ reported that the removal rate of chromium increase for initial concentrations of chromium in range 10 to 20 mg/L but decrease above this level due to binding sites saturation. The numerous number of chromium ions was competing with the binding sites of the algal biomass^42^. Sutkowy and Klosowski^43^ applied the alga *Pseudopediastrum* sp. as biosorpent of Cr (VI), they reported that the biosorption capacity when increasing of initial concentrations of the metal. Kumar et al.^44^ reported that increase of initial concentrations of Cr(VI) resulted in an increase in chromium sorption by filamentous algae that may be due to the accessibility of more surface area of the adsorbent. When the concentration of chromium increases, *Chlorella vulgaris* and *Scenedesmus acutus* remove the least amount of chromium despite increasing the driving force^45^. Zhang et al*.*^46^ reported that the chromium removal by activated carbon derived from algal bloom residue were decreased from 91.9 to 85.5% with increasing initial Cr(VI) concentration from 50 to 200 mg/L. The chromium removal capacity by brown alga *Dictyopteris polypodioides* decreased from 96.3 to 37.6%, when chromium concentration increased from 50 to 500 mg/L due to the binding sites saturation^47^. Li et al*.*^48^ investigated that the uptake of Cr(VI) by *Polysiphonia urceolata* was ranged from 16.1 to 128.2 mg/L and by *Chondrus ocellatus* was ranged from 17.3 to 105.2 mg/L when chromium concentrations varied from 25 to 250 mg/L, the increase of percentage removal may be due to increase of biosorbent doses. Katircioğlu et al*.*^49^ used *Oscillatoria* sp. as biosorbent for Cr (VI), and demonstrated that the chromium removal increased when the initial Cr(VI) concentration was increased from 25 to 200 mg/L.

### Effect of malachite green concentrations

The percentage of decolonization of malachite green by *Enteromorpha* was decreased with increase dye concentrations^31^. The removal percentage of malachite green by algal bloom residues decreased from 50.9 to 33.9% when the initial concentration of MG was increased from 50 to 100 mg/L^50^. The maximum decolonization of malachite green (71.41%) by *Pithopora* sp. was attained at an initial dye concentration of 15 ppm^51^. Maximum malachite green removal efficiency (73.49 and 91.61%) was attained by using dye concentrations of 6 mg/L by *Scenedesmus quadricauda* and *Chlorella vulgaris* biomass; respectively^52^. The highest removal of malachite green by *Chlorella, Cosmarium and Euglena* were obtained by increasing the initial dye concentration^53^. The removal of malachite green by brown alga *Laminaria japonica* decreased with increasing solution ionic strength^54^. Al-Fawwaz and Abdullah^55^ demonstrated that the efficiency of malachite green removal by immobilized *Desmodesmus* sp. increased from 63.2 to 89.1% as the initial dye concentrations increased from 5 to 20 mg/L; respectively.

### Effect of the initial pH

The results obtained have shown that the optimum pH affects the simultaneous removal of both MG and chromium ions. Dry cells of *Enteromopha* sp. consist of polysaccharides (63%), proteins (9.2%), lipids (13.8%) and ash content (1.4)^56^. *Enteromopha* cells composed of various functional groups such as carboxylic, hydroxyl, amines and amides. In an acidic solution, the functional groups were protonated and compete with the metal ions and dye, therefore in acidic solutions the biosorption efficiency decrease^57^. There is a direct relationship between negative charge and pH; an increase in pH causes an increase in negative charge of functional groups until all functional groups are deprotonated^58^. Data collected in Table 4 clear that the maximum removal of MG was at pH ranged from 5 to 10 when using different algae as adsorbent^52,54,57,59–65^. Also in agreement with study, the maximum removal of MG by algae *Sargassum crassifolium*, *Gracilaria corticata* and *Turbinaria conoides* was obtained at pH 8. On the other hand, the maximum removal of MG by *Ulva lactuca* was obtained at pH 7^65^. The maximum removal of the MG by *Sargassum swartzii* was obtained at pH 10^66^. According to the summarized data in Table 5, the optimum pH, initial chromium concentrations and also initial adsorbent concentrations vary according to the algae types^40,43–45,48,49,67–70^. With an increase in pH, the number of negatively charged binding sites increases, which would attract more cations (positive charge) of heavy metals or basic dye (MG)^71^. So in this study the optimum pH was 9.92 for simultaneous removal of MG and chromium ions.

**Table 4:** Optimization factors for removal of malachite green dye from aqueous solutions by various algae.

Adsorbent	Initial dye conc. (mg/L)	Algal doses (g/L)	pH	Contact time (min)	Removal efficiency (%)	References
*Scenedesmus quadricauda*	6	0.004	6	69	73.49	Kousha et al.^52^
*Chlorella vulgaris*	6	0.004	6	90	91.61	Kousha et al.^52^
*Laminaria japonica*	80	5	6	10	93.95	Wang et al.^54^
*Sargassum crassifolium*	5	1 g/L	8.0	60	95.6	Omar et al.^57^
*Ulva lactuca*	5	1 g/L	8.0	60	93.8	Omar et al.^57^
*Gracilaria corticata*	5	1 g/L	8.0	60	92.5	Omar et al.^57^
*Cosmarium* sp.	10	4.5 × 10^6^ cells mL^− 1^	9	210	92.4	Daneshvar et al.^59^
*Pandoraea pulmonicola* YC32	50	5	7–10	–	85.2	Chen et al.^60^
*Pithophora* sp.	100	0.015	5	50	95.14	kumar et al.^61^
*Turbinaria conoides*	100	3	8	225	66.6	Hameed and El-Khaiary^62^
*algal biomass*	80	0.02	2.9	60	100	Jasim and Abbas^63^
*Chlorella pyrenoidosa*	15	4 mL	7	6 days	95	Thirumagal and Panneerselvam^64^
*Ulva lactuca*	100	0.1 g/L	7	60	75.35	Deokar and Sabale^65^

**Table 5:** Optimization factors for removal of chromium from aqueous solutions by various algae.

Adsorbent	Initial chromium conc. (mg/L)	Algal doses (g/L)	pH	Contact time	Removal efficiency	References
*Chlorella sorokiniana*	100	–	7	24 h	99.6793%	Husien et al.^40^
*Pseudopediastrm boryanum*	10	2	2	15 min	70%	Sutkowy and Kłosowski^43^
Filamentous algae	10	0.250	2	70	17.24 mg/g	Kumar et al.^44^
*Chlorella vulgaris*	20	2.34	4.5	24 h	88.2%	Ardila et al.^45^
*Scenedesmus acutus*	20	2	5.34	24 h	87.1%	Ardila et al.^45^
*Polysiphonia urceolata*	250	4	2	60 min	170.6 mg/g	Li et al^48^
*Chondrus ocellatus*	250	4	2	40 min	113.4 mg/g	Li et al.^48^
*Oscillatoria limnetica*	200	1	6	60 min	15.81 mg/g	Katircioğlu et al.^49^
*Nostoc* sp*.*	100	0.2	5.4	120	29 mg/g	Coronel and Varela^68^
*Spirogyra porticalis*	40	1	3	60	70%	Elham and Sayyaf^69^
*Chlorella vulgaris*	100	1.2 g/L	3	60	99.75	Indhumathi et al.^70^

### Effect of the biosorbent dosage

In this study, the biosorbent dosage (*Enteromorpha* biomass concentration) affects the removal efficiency of both MG and chromium. The highest removal efficiency of both MG and chromium was obtained using 4.3 g/L of *Enteromorpha* biomass concentration. A highest removal of chromium was 66.6% when using 1.0 g of the dried alga, *Cladophora glomerata,* /100 mL aqueous solutions contains 20 mg/L chromium^32^. The highest chromium removal percentage (99.75%) by dry alga, *Chlorella vulgaris*, was obtained using 60 mg/50 mL solutions (1.2 g/L)^70^. Gandhi et al.^72^ demonstrated that the highest percentage uptake of chromium was obtained with 8.0 g algae as biosorbent. The highest chromium removal (83.55%) was obtained with 0.6 g/L *Sargassum* sp. after 120 min of contact time^73^. Whereas, highest chromium removal was obtained with 60 mg/L *Sargassum* sp. after 40 min of contact time^74^.

### Effect of contact (incubation) time

The maximum dye removal (91.92%) was obtained by using 1.25 g/L *U. lactuca* as biosorbent after 110 min of contact time^75^. The maximum removal of MG by *Scenedesmus* sp. MCC26 was obtained after 60 min of contact time^76^. Al-Homaidan et al.^32^ reported that the removal of chromium by green algae (*Microspora amoena*, *Enteromorpha intestinalis* and *Cladophora glomerata*) remain constant after one hour which indicated saturations. Gurbuz^77^ noticed that the removal of Cr(VI) ions when using green alga *Scenedesmus* as biosorbent was quick during the first 30 min (65.62 ± 2.4%), then increase to 92.7 ± 4.12% after 1 h. Sala et al*.*^78^ reported that the maximum removal of chromium ions (60%) by dried marine alga *Sargassum* sp. was obtained in ten min.

### Optimization using the desirability function

**Figure 4:**
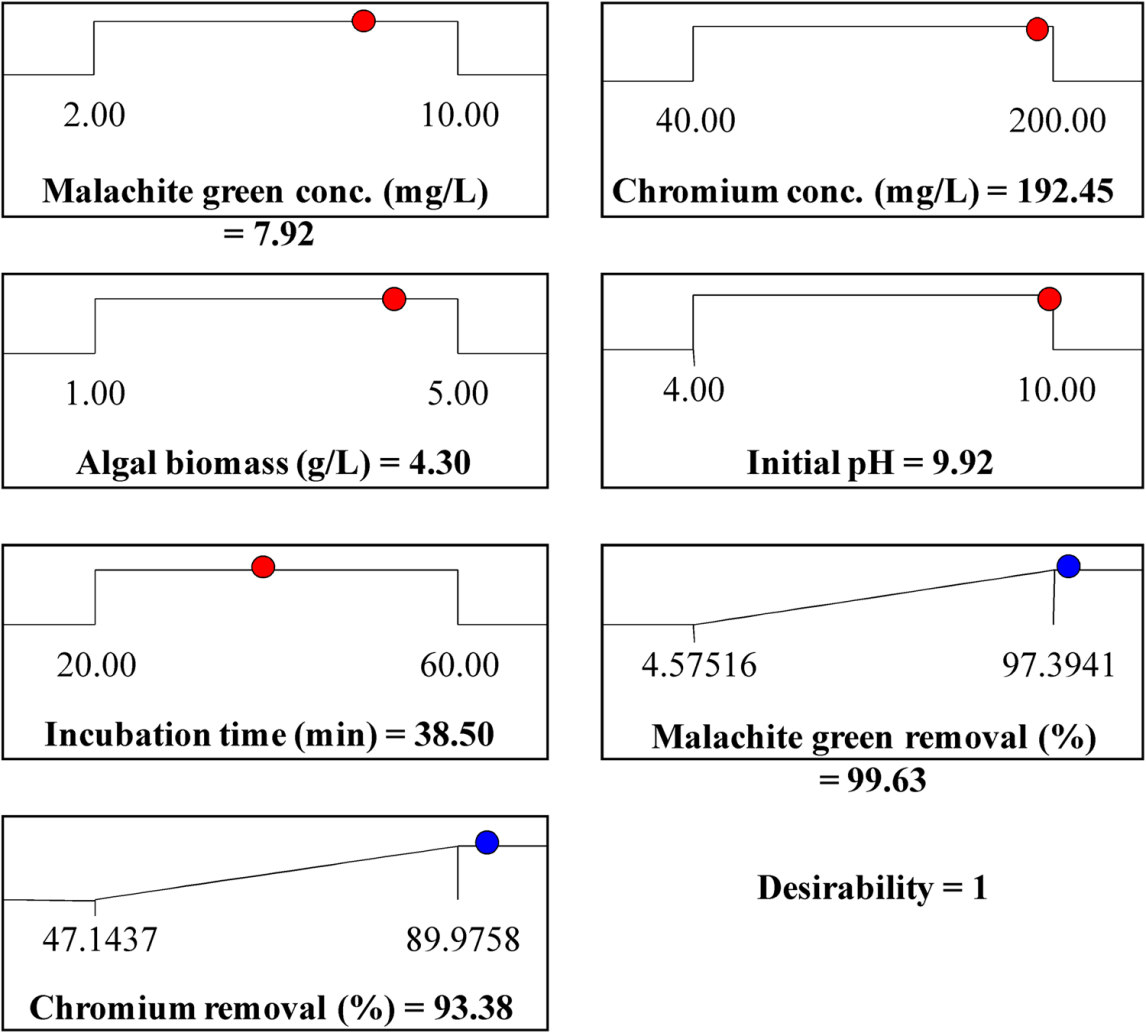
The desirability function and the optimum predicted values for the maximum adsorption of malachite green and chromium. Image was created by using Design Expert version 7 for Windows software.

Design Expert software was used for optimization to identify the best working conditions for the highest simultaneous malachite green and chromium ions removal. The program's desirability function has been set from zero to one for each factor. The maximization of this desirability function is the ultimate objective of this program. Due to the curvature format of the response surfaces, more than one maximum point is expected, and their combinations into the desirability function. This software begins in the design space from many points, until the search completes by finding the best maximum for the responses^79,80^. Figure 4 shows the desirability values of the numerical optimization to find the optimum points which maximizes the removal % of both malachite green removal and chromium ions. Figure 4 shows that the maximum predicted malachite green removal and chromium removal could be 99.63 and 93.38%; respectively by using malachite green concentrations of 7.92 mg/L, chromium concentrations of 192.45 mg/L, algal biomass of 4.30, pH of 9.92 and contact time for 38.5 min. These optimum values were verified experimentally which resulted in malachite green removal of 99.4% and chromium removal of 94.17%.

### FTIR analysis

**Figure 5:**
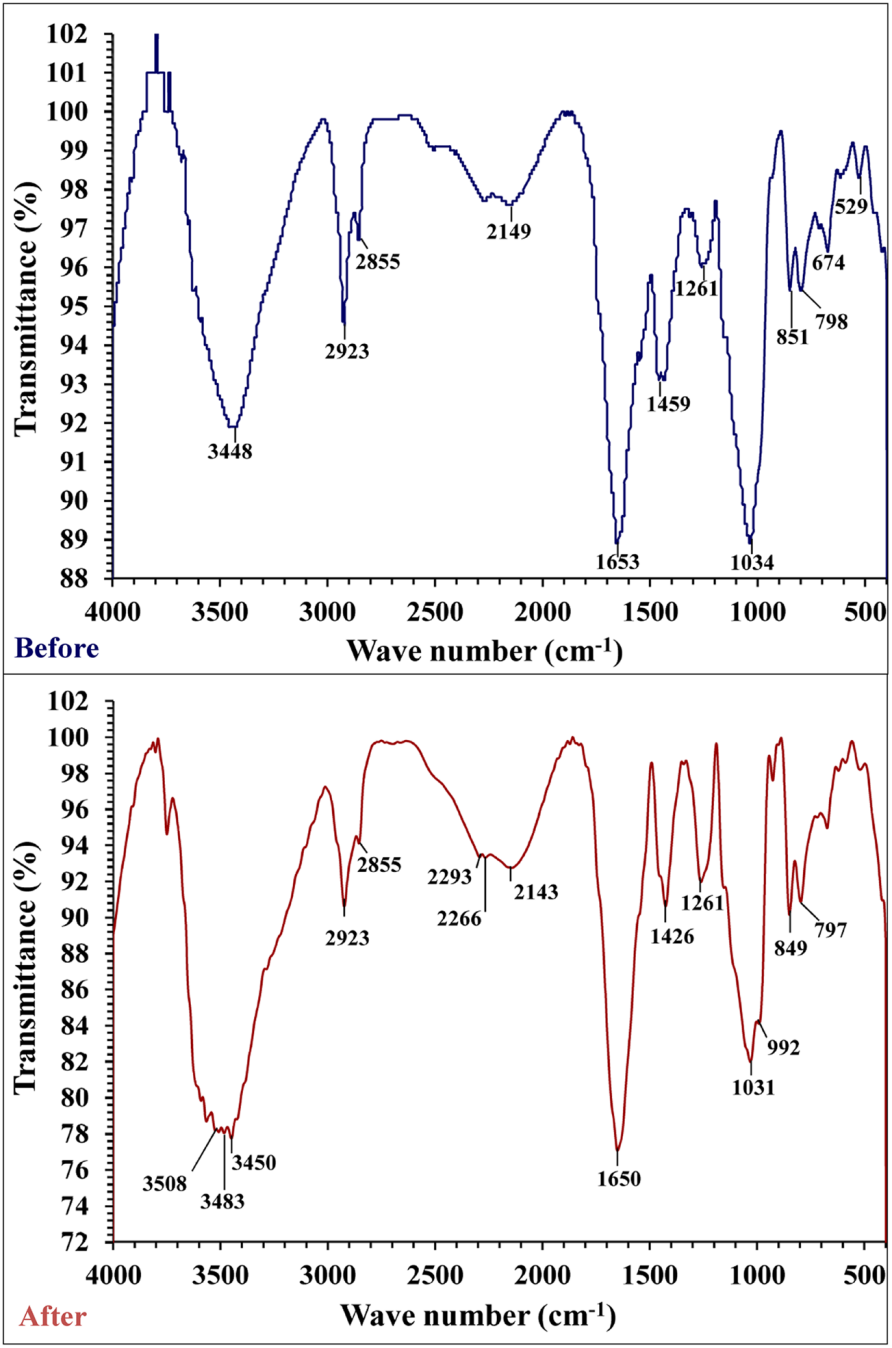
FTIR of *E. intestenalis* biomass: (before chromium and MG biosorption and after chromium and MG biosorption).

The FTIR spectrums of *E. intestinalis* biomass samples were analyzed before and after biosorption of malachite green and chromium (Table 6, Fig. 5) to notice any differences because of the interaction of dye and metal ions with binding sites (functional groups) that occurs on the biomass cell surface. “The macro green alga cell walls, consist of the major content of polysaccharides, and have many functional groups which carrying negative charges that can interact with cationic dye and bind heavy metal ions, and these functional groups include carboxylate, hydroxyl, amino and phosphate groups^81^. The spectra of adsorbents before and after treatments were measured within the range of 400–4,000 cm^−1^ wave number”^82^ .The spectrum of FTIR analysis of *E. intestinalis* before and after biosorption of chromium and malachite green showed different absorption peaks at 3,448, 2,923, 2,855, 2,149, 1,653, 1,459, 1,261, 1,034, 851, 798, 674 and 529 cm^−1^ which has been shifted to 3,508, 3,483, 3,450, 2,923, 2,855, 2,266, 2,143,1,650,1,426, 1,261, 1,031, 992, 849 and 797 cm^−1^ respectively. The broad peak in the pure biomass of *E. intestinalis* before malachite green dyes and chromium biosorption at 3,448 is assigned to alcohols (O–H) groups^83^. The peaks at 2,923 and 2,855 cm^–1^ are related to (C–H stretching)^84^. The peaks ranged from 2014 to 2,162 cm^−1^ is due to C=C from alkynes and the peak at 2,149 cm^−1^ is related to alkynes^85^. Peak at 1653 cm^−1^ is due to carbonyl group as observed by Muinde et al*.*^85^. The peaks between 1629.45 and 1732.02 cm^−1^ are characteristic of carbonyl group. Peaks demonstrated –CH_3_ stretch can be observed at 1,459 cm^−1^^86^. Peaks at 1,261 cm^−1^ restricted to C–O stretching^87^. The peaks at 1,034 cm^-1^ correspond to the C–N stretching mode^88^. Peak at 851 referred to C(1)–H(α) bending^89^. Peaks ranged from 900 to 675 (s) assigned to C–H “oop” aromatics^90^. After the malachite green and heavy metals absorption the wavenumber of the peaks are shifted to higher or less wavenumber. The –OH absorption peak at 3,448 cm^−1^ is shifted to 3,508, 3,483 and 3,450 cm^−1^. There are two small peaks at 2,293 cm^−1^ and 2,266 cm^−1^ observed in the FT-IR spectroscopy curve after absorption of malachite green and chromium; these peaks may due to alkanes^91^. Figure 5 demonstrated that peaks 1653, 1,459, 1,034, 851 and 798 cm^−1^ are shifted to 1,650, 1,426, 1,261, 1,031, 849 and 797 cm^−1^. These shifted absorption peaks could be attributable to chemical bonding among binding sites on algal biomass and the malachite green dyes, or chromium^92^. The small difference between the wave number of peaks after and before treatments with simultaneously malachite green and chromium it is presumed that the dye and heavy metals incorporated within the adsorbent through interaction with the active functional groups^31^.

**Table 6:** FTIR of *E. intestenalis* biomass: (before and after chromium and MG biosorption) summary of wave numbers and corresponding functional groups.

Before adsorption	After adsorption	Functional groups
3,448	3,508–3,483-3,450	3,600–2,800 Sharp peak (Alcohol or Phenol free OH) ν(H-bonded OH) Carboxylic acid: very broad peak
2,923, 2,855	2,923, 2,855	C–H stretching
–	2,293–2,266	alkanes
2,149	2,143	2,260–2,100 C≡ C stretch (alkynes)
1653	1,650	1,680–1,640–C=C– stretch (alkenes)
1,459	1,426	1,500–1,400 (m) C–C stretch (in–ring) aromatics
1,261	1,261	1,300–1,150 (m) C–H wag (CH_2_ X) alkyl halides
1,034	1,031	1,250–1,020 (m) C–N stretch aliphatic amines
–	992	CH_2_
851	849	900–675 (s) C–H "oop" aromatics
798	797	800–600 C–Cl
674	–	
529	–	750–500 C-I

### Scanning electron microscopy (SEM)

**Figure 6:**
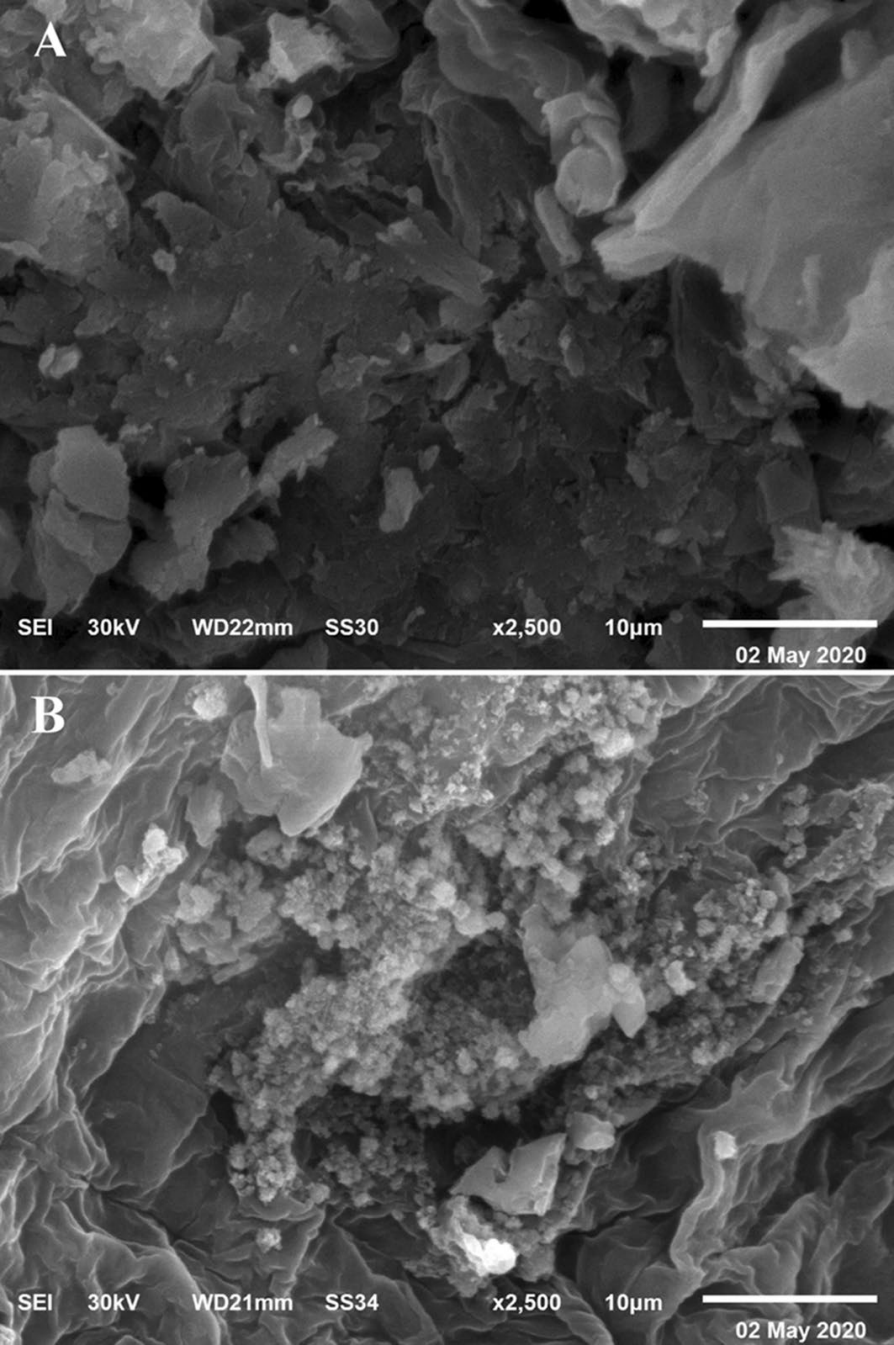
SEM micrograph of *E. intestinalis* biomass: (**A**) before and (**B**) after adsorption of MG and chromium from aqueous solution.

Figure 6A,B shows that SEM micrograph of *E. intestinalis* biomass after and before malachite green and chromium adsorption. The results investigated that the control alga is relatively smooth surface and a little amount of impurities was present, whereas the treated alga had a rough surface and present a large amount of impurities may be due to MG and chromium absorbed on the alga surface. The cell wall of *Sargassum swartzii* after biosorption of MG appeared shrinkage in comparison to alga before absorption MG^66,93^. The rough surface with micropores of *Chlorella vulgaris* particles was showed under SEM after absorption of chromium^70^.

## Material and methods

### Collection and preparation of biosorbent

*E. intestinalis* was collected from Jeddah, Saudi Arabia beach on April 2019, and was identified according to Taylor^94^. The *E. intestinalis* biomass were washed thoroughly under running tap water, and then distilled water to completely remove salts and sand. The cleaned marine green alga biomass was dried in oven at 60 °C, until the moisture was completely removed (up to constant weight). Furthermore, the dried alga biomass was milled and the grounded powder was sieved using the standard laboratory test sieve. The ground alga biomass with particle size range of 1–1.2 mm was used as biosorbent for biosorption experiments for simultaneously malachite green and chromium removal in the present study^8^.

### Preparation of malachite green and heavy metal solutions

The required solutions used for the biosorption experiments were prepared. The initial concentrations of Cr(VI) ions (40, 120, 200 mg/L) and malachite green (2, 6, 10 mg/L) were prepared by dissolving known quantity of potassium dichromate or malachite green in 1 L distilled water^95,96^. The initial pH level of each solution was adjusted using 0.1 N HCL and 0.1 N NaOH to the desired level.

### Statistical optimization of chromium and Malachite green biosorption by face centered central composite design (FCCCD)

The biosorption experiments were conducted in batch condition at room temperature (28 ± 2°C). The biosorption experiments were carried out in a series of 250 mL Erlenmeyer flask using FCCCD to evaluate the impacts of five variables and to determine their optimal levels on the chromium and MG biosorption. Fifty experimental trials which are shown in Table 1 were conducted with 8 runs at the midpoint for five process variables and each variable varies in three levels: − 1 (low level), 0 (standard, middle or zero level) and + 1 (high level). The chosen independent variables were initial concentration of MG (X_1_; 4, 6, 10 mg/L), initial concentration of Cr(VI) (X_2_; 40, 120, 200 mg/L), biosorbent concentration (X_3_; 1, 3, 5 g/L), initial pH level (X_4_; 4, 7, 10) and contact time (X_5_; 20, 40, 60 min) at a constant agitation speed (200 rpm).

The relationships between the five independent process variables and the responses (% Cr(VI) and MG biosorption) were determined using the second-degree polynomial equation as follows:

3 $$Y = \beta_{0} + \sum\limits_{i} {\beta_{i} X_{i} + \sum\limits_{ii} {\beta_{ii} X_{i}^{2} } } + \sum\limits_{ij} {\beta_{ij} X_{i} X_{j} }$$

In which Y is the predicted Cr(VI) or MG biosorption perecntage, the linear coefficient (β_i_), quadratic coefficients (β_ii_), the regression coefficients (β_0_), the interaction coefficients (β_ij_) and the coded values of the independent variables (X_i_).

### Statistical analysis

Design Expert version 7 for Windows software was used for the experimental designs and statistical analysis. The statistical software package, STATISTICA software (Version 8.0, StatSoft Inc., Tulsa, USA) was used to plot the three-dimensional surface plots.

### Analytical methods

Ten milliliters of the binary solution for each trial of FCCCD was centrifuged, and the supernatants were analyzed using the spectrophotometer by measuring the absorbance changes at a wavelength of λ_max_ 616 nm to determine the final (residual) concentrations (Cf) of malachite green dye. The efficiency of *E. intestinalis* biomass for malachite green removal from aqueous solutions was determined in percentage using the following equation:

4 $${\text{Malachite green removal}}\; (\% ) = \frac{{{\text{C}}_{{\text{i}}} - {\text{C}}_{{\text{f}}} }}{{{\text{C}}_{{\text{i}}} }} \, \times 100$$

where: C_i_, C_f_ are the initial and final malachite green concentrations (mg/L); respectively.

Another 10 mL of the binary solution for each trial were analyzed to determine the residual concentration of Cr(VI) ions using Atomic absorptions (Buck scientific 2 Accusystem series Atomic Absorption (USA) by an air acetylene system) in the Biotechnology Unit, Mansoura university Egypt^97^. The efficiency of *E. intestinalis* biomass for chromium ions elimination from aqueous solutions was determined in percentage using the following equation:

5 $${\text{Chromium ions removal (\%) = }}\frac{{{\text{C}}_{{\text{i}}} - {\text{C}}_{{\text{f}}} }}{{{\text{C}}_{{\text{i}}} }} \, \times 100$$

where: C_i_, C_f_ are the initial and final chromium ions concentrations (mg/L); respectively.

All determinations of both chromium ions and malachite green in the binary solution were estimated in triplicates.

### Fourier transform infrared (FTIR) spectroscopy

The FTIR spectroscopy is a significant tool used to identify the distinctive functional groups that may be responsible for the biosorption process of both malachite green and chromium ions by the surface of *E. intestinalis* biomass. The dry biomass of *E. intestinalis* samples were analyzed using FTIR spectroscopy before and after malachite green and chromium ions removal. The samples of dry biomass were mixed with pellets of potassium bromide and the FTIR spectra were then determined within the range of 400–4,000 cm^−1^ using “Thermo Fisher Nicolete IS10, USA spectrophotometer”.

### Scanning electron microscopy (SEM)

The samples of *E. intestinalis* dry biomass were investigated after and before chromium and malachite green removal using SEM to examine the cell surface morphology of *E. intestinalis* biomass before and after the biosorption process of both chromium and malachite green. The gold-coated dry biomass samples were investigated at various magnifications using accelerated beam voltage of 30 keV.

## Conclusions

This study presents a novel approach that uses macro-green algae, *Enteromorpha intestinalis*, to remove both MG dye and chromium ions simultaneously from aqueous solutions. Maximum experimentally verified malachite green removal and chromium removal were 99.4 and 94.17%; respectively by using malachite green concentrations of 7.92 mg/L, chromium concentrations of 192.45 mg/L, algal biomass of 4.30, pH of 9.92 and contact time for 38.5 min. *E. intestinalis* dry biomass can be used as an effective and affordable biosorbent for the removal of MG and chromium ions from waste water, and the procedure used is safe and environmentally friendly.

## Supplementary information

Supplementary Information
