# Author Correction: Bioprocessing strategies for cost-effective simultaneous removal of chromium and malachite green by marine alga *Enteromorpha intestinalis*

**DOI:** 10.1038/s41598-020-72424-6

**Published:** 2020-09-23

**Authors:** Ragaa A. Hamouda, Noura El-Ahmady El-Naggar, Nada M. Doleib, Amna A. Saddiq

**Affiliations:** 1 Department of Biology, Faculty of Sciences and Arts Khulais, University of Jeddah, Jeddah, Saudi Arabia; 2 Microbial Biotechnology Department, Genetic Engineering and Biotechnology Research Institute, University of Sadat City, Sadat City, Egypt; 3 Department of Bioprocess Development, Genetic Engineering and Biotechnology Research Institute, City of Scientific Research and Technological Applications (SRTA-City), Alexandria, Egypt; 4 Department of Microbiology, Faculty of Applied and Industrial Science, University of Bahri, Khartoum, Sudan; 5 Department of Biology, Faculty of Sciences, University of Jeddah, Jeddah, Saudi Arabia

Correction to: *Scientific Reports* 10.1038/s41598-020-70251-3, published online 10 August 2020.

The original version of this Article contained an error in Affiliation 3, which was incorrectly given as ‘Department of Bioprocess Development, Genetic Engineering and Biotechnology Research Institute, City of Scientific Research and Technological Applications, Alexandria, Egypt’. The correct affiliation is listed below:

Department of Bioprocess Development, Genetic Engineering and Biotechnology Research Institute, City of Scientific Research and Technological Applications (SRTA-City), Alexandria, Egypt.

This error has now been corrected in the HTML and PDF versions of the Article, and in the accompanying Supplementary Information.

