# Supplementary material for: Bioprocessing strategies for cost-effective simultaneous removal of chromium and malachite green by marine alga Enteromorpha intestinalis
Source: Sci Rep. 2020 Aug 10;10:13479. doi: 10.1038/s41598-020-70251-3 (PMC7417574; doi:10.1038/s41598-020-70251-3)
Supplement: Supplementary file 1 — Supplementary Information [file 41598_2020_70251_MOESM1_ESM.docx]

**Bioprocessing strategies for cost-effective simultaneous removal of chromium and malachite green by marine alga *Enteromorpha intestinalis***

**Ragaa A. Hamouda^1,2^,** ***Noura El-Ahmady El-Naggar^3^, Nada M. Doleib^1,4^**, **Amna A. Saddiq^5^**

^1^Department of Biology, Faculty of sciences and Arts Khulais, University of Jeddah, Jeddah Saudi Arabia

^2^Microbial Biotechnology Department, Genetic Engineering and Biotechnology

Research Institute, University of Sadat City, Egypt

^3^Department of Bioprocess Development, Genetic Engineering and Biotechnology Research Institute, City of Scientific Research and Technological Applications, Alexandria, Egypt.

^4^Department of Microbiology, Faculty of Applied and Industrial Science, University of Bahri, Khartoum, Sudan

^5^ Faculty of Sciences, Department of Biology, University of Jeddah, Jeddah, Saudi Arabia

**Corresponding Author’s information**

**Dr. Noura El-Ahmady Ali El-Naggar**

**Address:**

Bioprocess Development Department,

Genetic Engineering and Biotechnology Research Institute,

City of Scientific Research and Technological Applications,

New Borg El- Arab City, 21934, Alexandria, Egypt

**Tel:** (002)01003738444

**Fax:** (002)03 4593423

**E-mail:** [nouraalahmady@yahoo.com](mailto:nouraalahmady@yahoo.com)

**Supplementary Table 1.** Fit summary for FCCCD results of malachite green adsorption by [*E. intestinalis*](https://www.google.com.sa/search?safe=strict&sxsrf=ALeKk01cyHE3FkyuYMrgj_G0T-R5wwTOgA:1584112242355&q=Enteromorpha+intestinalis&spell=1&sa=X&ved=2ahUKEwiIrfX73ZfoAhVqMewKHeUQA-4QkeECKAB6BAgUEC0)

| **Lack of Fit Tests** | | | | | | | | |
| --- | --- | --- | --- | --- | --- | --- | --- | --- |
| **Source** | **Sum of Squares** | ***df*** | **Mean Square** | | ***F-*value** | | ***P-*value**  ***P*rob >*F*** | |
| Linear | 11993.53 | 37 | 324.15 | | 48.40 | | < 0.0001* | |
| 2FI | 9969.09 | 27 | 369.23 | | 55.13 | | < 0.0001* | |
| Quadratic | 444.75 | 22 | 20.22 | | 3.02 | | 0.0688 | |
| Pure Error | 46.88 | 7 | 6.70 | |  | |  | |
| **Sequential Model Sum of Squares** | | | | | | | | |
| **Source** | **Sum of Squares** | ***df*** | **Mean Square** | | ***F-*value** | | ***P-*value**  ***P*rob >*F*** | |
| Linear vs Mean | 31728.98 | 5 | 6345.80 | | 23.19 | | < 0.0001* | |
| 2FI vs Linear | 2024.44 | 10 | 202.44 | | 0.69 | | 0.7288 | |
| Quadratic vs 2FI | 9524.34 | 5 | 1904.87 | | 112.36 | | < 0.0001* | |
| Residual | 230.66 | 14 | 16.48 | |  | |  | |
| **Model Summary Statistics** | | | | | | | | |
| **Source** | **Standard deviation** | **R-Squared** | | **Adjusted R-Squared** | | **Predicted R-Squared** | | **PRESS** |
| Linear | 16.54 | 0.7249 | | 0.6937 | | 0.6687 | | 14502.89 |
| 2FI | 17.16 | 0.7712 | | 0.6702 | | 0.6283 | | 16266.98 |
| Quadratic | 4.12 | 0.9888 | | 0.9810 | | 0.9642 | | 1568.50 |
| * Significant values,  *df* : degree of freedom, PRESS: sum of squares of prediction error, two factors interaction: 2FI | | | | | | | | |

**Supplementary Table 2.** Fit summary for FCCCD results of chromium adsorption by [*E. intestinalis*](https://www.google.com.sa/search?safe=strict&sxsrf=ALeKk01cyHE3FkyuYMrgj_G0T-R5wwTOgA:1584112242355&q=Enteromorpha+intestinalis&spell=1&sa=X&ved=2ahUKEwiIrfX73ZfoAhVqMewKHeUQA-4QkeECKAB6BAgUEC0).

| **Lack of Fit Tests** | | | | | | | | |
| --- | --- | --- | --- | --- | --- | --- | --- | --- |
| **Source** | **Sum of Squares** | ***df*** | **Mean Square** | | ***F-*value** | | ***P-*value**  ***P*rob >*F*** | |
| Linear | 2274.57 | 37 | 61.47 | | 61.90 | | < 0.0001* | |
| 2FI | 1258.58 | 27 | 46.61 | | 46.94 | | < 0.0001* | |
| Quadratic | 41.74 | 22 | 1.90 | | 1.91 | | 0.1927 | |
| Pure Error | 6.95 | 7 | 0.99 | |  | |  | |
| **Sequential Model Sum of Squares** | | | | | | | | |
| **Source** | **Sum of Squares** | ***df*** | **Mean Square** | | ***F-*value** | | ***P-*value**  ***P*rob >*F*** | |
| Linear vs Mean | 4481.84 | 5 | 896.37 | | 17.29 | | < 0.0001* | |
| 2FI vs Linear | 1015.99 | 10 | 101.60 | | 2.73 | | 0.0140* | |
| Quadratic vs 2FI | 1216.84 | 5 | 243.37 | | 144.94 | | < 0.0001* | |
| Residual | 15.96 | 14 | 1.14 | |  | |  | |
| **Model Summary Statistics** | | | | | | | | |
| **Source** | **Standard deviation** | **R-Squared** | | **Adjusted R-Squared** | | **Predicted R-Squared** | | **PRESS** |
| Linear | 7.20 | 0.6627 | | 0.6243 | | 0.5706 | | 2904.03 |
| 2FI | 6.10 | 0.8129 | | 0.7303 | | 0.7392 | | 1763.71 |
| Quadratic | 1.30 | 0.9928 | | 0.9878 | | 0.9754 | | 166.15 |
| * Significant values,  *df* : degree of freedom, PRESS: sum of squares of prediction error, two factors interaction: 2FI | | | | | | | | |

**Supplementary Figure 1. Malachite green structure**
